# Bone Tumor Microenvironment Is Associated with Meningioma Enlargement and Inflammatory Transcriptional States: A Candidate Role for Macrophage-Derived IL-1β

**DOI:** 10.3390/biomedicines14092019

**Published:** 2026-09-08

**Authors:** Kazushi Suzuki, Takanobu Kabasawa, Takumi Kitaoka, Naoya Uchiyama, Naing Ye Aung, Mitsuru Futakuchi

**Affiliations:** Department of Pathology, Faculty of Medicine, Yamagata University, Yamagata 990-9585, Japan

**Keywords:** meningioma, bone involvement, xenograft model, tumor microenvironment, tumor–stromal interaction, RNA-seq, ligand–target interaction analysis

## Abstract

**Background/Objectives:** Meningiomas frequently involve the adjacent skull bone. The bone tumor microenvironment (TME) represents a unique niche in bone-invasive tumors and bone metastases, and can promote tumor progression through tumor–stromal interactions. We hypothesized that the bone TME may contribute to tumor cell transcriptional changes and tumor growth in meningioma. **Methods:** Human malignant meningioma cell lines, IOMM-Lee and HKBMM, were implanted over the calvarial bone (bone TME) and into the dorsal skin (non-bone TME) of BALB/c-nu/nu mice. Tumors from the IOMM-Lee model underwent bulk RNA sequencing with computational separation of human- and mouse-derived reads, enabling ligand–target interaction analysis of stromal cell-to-tumor cell signaling. Differential gene expression analysis, enrichment analysis, ligand–target interaction analysis, multiplex immunofluorescence, and immunohistochemistry were performed. **Results:** Tumor volume was significantly greater in the bone TME than in the non-bone TME in both models, whereas the Ki-67 labeling index was significantly reduced. Transcriptomic analysis revealed distinct tumor cell transcriptional states in the bone TME, characterized by upregulation of inflammation-related genes and enrichment of inflammatory signaling pathways, including interferon responses and NF-κB signaling. Ligand–target interaction analysis of stromal cell-to-tumor cell signaling identified stromal *IL1B* as a candidate regulator of inflammatory transcriptional programs in tumor cells. F4/80-positive macrophages expressed IL-1β, and tumor cells were positive for phospho-IKKα/β and phospho-IκBα in the bone TME. **Conclusions:** Tumor–stromal interactions potentially involving macrophage-derived IL-1β may be associated with inflammatory transcriptional programs in meningioma cells and with meningioma enlargement in the bone TME. These interactions may be therapeutically relevant in meningioma with bone involvement.

## 1. Introduction

Meningioma is the most common primary intracranial tumor. Meningiomas are mostly benign, and complete surgical resection can lead to cure [1,2]. However, in meningiomas arising in anatomically challenging locations such as the skull base, or those involving adjacent tissues, gross total resection is often difficult to achieve [1,3]. Because the extent of surgical resection is known to be associated with recurrence risk and patient prognosis, these features represent important clinical challenges in meningioma management [1,2,4]. Although radiotherapy is commonly used for unresectable meningiomas, durable tumor control remains difficult in some cases, and no effective systemic therapy is currently established [5,6].

Meningiomas can involve the adjacent skull bone and induce extensive bone remodeling, including hyperostosis and osteolytic changes [7,8]. Several studies have reported that bone involvement itself has not been consistently associated with tumor progression or recurrence in meningioma [7,9]. However, other studies have reported that bone involvement is associated with increased surgical difficulty and lower rates of gross total resection [1,10]. Therefore, bone involvement can indirectly contribute to poorer clinical outcomes by limiting complete tumor removal [1,10]. Recent studies have highlighted the importance of the bone tumor microenvironment (TME) in meningioma, suggesting that tumor cells actively interact with microenvironmental cells rather than merely invading bone as a passive anatomical structure [3,11].

The TME is known to play a critical role in tumor progression through dynamic interactions between tumor cells and stromal cells [12,13]. In particular, the bone TME has been recognized as a unique niche in bone-invasive tumors and bone metastases, including those of prostate cancer [14,15], breast cancer [16], and renal cell carcinoma [17]. The bone TME comprises diverse cell types, including macrophages and other immune cells, fibroblasts, osteoclasts, and osteoblasts, as well as various cytokines and growth factors. The bone TME has also been shown to promote tumor progression through tumor–stromal interactions [14,15,16,17].

In various cancers, such as breast cancer [18], colorectal cancer [19], and oral squamous cell carcinoma [20], tumor–stromal interactions have been shown to alter tumor cell phenotypes. In brain-invasive meningioma, a recent study demonstrated that interactions between tumor cells and brain parenchymal stromal cells change tumor cell transcriptional states and promote meningioma growth [21]. In these interactions, various signaling molecules produced by stromal cells, including inflammatory cytokines, chemokines, and other soluble factors, can activate multiple signaling pathways in tumor cells. These changes modulate tumor cell proliferation, invasion, immune evasion, and therapy resistance [12,13,18,19,21]. Recent advances in transcriptomic analysis have further highlighted the importance of ligand–target–receptor interactions between tumor cells and stromal cells in tumor cell transcriptional reprogramming [22,23].

Here, we hypothesized that the bone TME may contribute to tumor cell transcriptional changes and tumor growth in meningioma. In this study, we evaluated the effects of the bone TME on meningioma growth by comparing tumors implanted in the bone and non-bone microenvironments using a mouse xenograft model. Because human tumor cell-derived and mouse stromal cell-derived reads can be computationally distinguished in xenograft tumors, this model enables us to analyze tumor cell transcriptional programs and to infer the direction of stromal cell-to-tumor cell signaling within the TME from bulk RNA sequencing (RNA-seq) data. By combining species-specific read separation with ligand–target interaction analysis, we characterized transcriptional changes in tumor cells and explored stromal cell-derived signaling associated with the bone TME. Our findings highlight the importance of tumor–stromal interactions within the bone TME of meningioma and may contribute to the development of novel therapeutic strategies for meningioma with bone involvement.

## 2. Materials and Methods

### 2.1. Cell Culture

IOMM-Lee is a cell line established from a human anaplastic intraosseous meningioma [24], and HKBMM is also a cell line established from a human malignant meningioma [25]. Both cell lines have shown reproducible tumor-forming capacity in nude mice, and have been used to establish orthotopic and heterotopic malignant meningioma xenograft models [24,25,26]. Bone-involving growth has also been documented in an orthotopic xenograft model [26]. In this study, we therefore used these cell lines to examine meningioma behavior in the bone-associated microenvironment. IOMM-Lee and HKBMM were obtained from the American Type Culture Collection (ATCC, Manassas, VA, USA; #CRL-3370) and the RIKEN BioResource Research Center (Tsukuba, Japan; #RCB0680), respectively. IOMM-Lee cells were cultured as a monolayer in Dulbecco’s Modified Eagle Medium (DMEM) supplemented with 10% fetal bovine serum (FBS) and 1% penicillin-streptomycin. HKBMM cells were cultured as a monolayer in Ham’s F-12 medium supplemented with 15% FBS and 1% penicillin-streptomycin. The cultures were maintained at 37 °C in a humidified atmosphere containing 5% CO_2_.

### 2.2. Animal Studies

IOMM-Lee or HKBMM cells (1–2 × 10^6^) were suspended in 100 μL of culture medium mixed with 100 μL of Matrigel (Corning Inc., Corning, NY, USA). The 200 μL cell suspension was subcutaneously injected over the calvarial bone and into the dorsal skin of 6- to 8-week-old female BALB/c-nu/nu mice (CLEA Japan Inc., Tokyo, Japan). For calvarial implantation, the cell suspension was injected percutaneously into the subcutaneous tissue of the occipital supraperiosteal region of the calvarial bone. The calvarial bone was not intentionally penetrated or mechanically disrupted during implantation. Following injection, the Matrigel-containing suspension gelled locally, limiting dispersion and retaining the cells as a localized mass over the calvarial bone. For dorsal implantation, the same cell suspension was injected percutaneously into the dorsal subcutaneous tissue. This model was a dual-site heterotopic xenograft model and was designed to examine the impact of the bone-associated microenvironment on meningioma behavior while minimizing the influence of brain parenchyma and other intracranial tissues. The “bone TME” denotes the subcutaneous calvarial implantation site, where tumor cells directly interact with the bone microenvironment. The “non-bone TME” denotes the dorsal subcutaneous implantation site without direct interaction with the bone microenvironment. Tumor volume at the implantation sites and body weight were measured twice weekly. Tumor volume was calculated using the following formula: (1/2) × (major axis [mm]) × (minor axis [mm])^2^. Mice were housed under specific pathogen-free conditions with controlled temperature and humidity with free access to food and water. Mice were euthanized by exsanguination under deep anesthesia and tumors were harvested from both implantation sites at 3 weeks (IOMM-Lee) and 7 weeks (HKBMM) after implantation, when tumors had reached a comparable size sufficient for subsequent analyses. Each tumor sample was divided into two portions, with one half used for histological analysis and the other for RNA extraction. For histological analysis, the harvested tissues were fixed in 10% neutral-buffered formalin at room temperature and embedded in paraffin. Tumor tissues containing calvarial bone were subsequently decalcified in a 10% ethylenediaminetetraacetic acid disodium salt solution. All animal studies were performed in accordance with the protocol approved by the Animal Research Committee of Yamagata University (approval number: R6148, 9 December 2024).

### 2.3. Immunohistochemistry (IHC) and Multiplex Immunofluorescence (IF) Studies

All IHC studies were performed on a BOND RX autostainer (Leica Biosystems, Nussloch, Germany) as previously described [18,19]. Positive reactions were detected as brown coloration with 3,3′-diaminobenzidine (DAB; BOND Polymer Refine Detection, Leica Biosystems). Multiplex IF staining was performed using the tyramide signal amplification-based Opal method with an Opal 4-Color Automation IHC Kit (Akoya Biosciences, Marlborough, MA, USA). Opal reagents (Opal520, FITC; Opal570, TRITC, Akoya Biosciences, Marlborough, MA, USA) were used for fluorescence detection instead of DAB. The sections were counterstained with spectral DAPI. The following primary antibodies were used in this study: Ki-67 (D3B5, Cell Signaling Technology, Danvers, MA, USA), IL-1β (D3H1Z, Cell Signaling Technology), F4/80 (D2S9R, Cell Signaling Technology), phospho-IKKα/β (16A6, Cell Signaling Technology), and phospho-IκBα (1G21, Proteintech, Chicago, IL, USA).

### 2.4. Image Analysis

Image analysis was performed using HALO software (v3.2, Indica Labs, Corrales, NM, USA) as previously described [18,19]. IHC slides were converted into digital whole-slide images using a NanoZoomer digital scanner (Hamamatsu Photonics, Hamamatsu, Japan) for analysis. Ten non-necrotic areas (each 0.1 mm^2^, square or rectangular) were randomly selected on each slide, and the ratio of Ki-67-positive cells to the total number of cells was calculated for each area using HALO. The average of these values was used as the Ki-67 labeling index for each tumor.

### 2.5. RNA Sequencing and Data Analysis

Total RNA was extracted from fresh tumor tissues using ISOGEN (NIPPON GENE, Tokyo, Japan) according to the manufacturer’s instructions. RNA concentration and purity were measured using a NanoVue spectrophotometer (Biochrom, Holliston, MA, USA), and RNA integrity was assessed using a TapeStation system (Agilent Technologies, Santa Clara, CA, USA). Samples with sufficient RNA quality were used for subsequent RNA-seq procedures. RNA library preparation and sequencing were performed by Rhelixa (Tokyo, Japan). Strand-specific poly(A)-selected RNA libraries were prepared from total RNA using the NEBNext Poly(A) mRNA Magnetic Isolation Module and NEBNext Ultra II Directional RNA Library Prep Kit, New England Biolabs, Ipswich, MA, USA. The libraries were sequenced on an Illumina NovaSeq X Plus platform to generate 150 bp paired-end reads. Approximately 40 million reads, corresponding to 20 million read pairs, were obtained per sample. After sequencing quality was assessed using FastQC (v0.12.1), adapter sequences and low-quality reads were trimmed using fastp (v0.23.2). The sequence data obtained from the xenograft mouse model contain reads derived from both human and mouse genes. To distinguish between human- and mouse-derived reads, the data were aligned separately to the human (GRCh38/hg38) and mouse (GRCm39/mm39) reference genomes using STAR (v2.7.10b) [27]. Subsequently, human- and mouse-specific reads were extracted using the XenofilteR R package (v1.6) [28] to avoid false positives arising from cross-species mapping. Gene expression counts were quantified by HTSeq (v2.0.9) for each species. The proportion of reads assigned to human and mouse genomes was calculated based on raw counts. Differential gene expression between tumors from the bone and non-bone TMEs was analyzed and count normalization were performed using the DESeq2 R package (v1.50.2) [29]. Principal component analysis (PCA) was performed using variance-stabilized normalized expression data obtained from DESeq2. Volcano plots were generated using differential gene expression analysis results obtained from DESeq2. For heatmap visualization, the top 50 differentially expressed genes (DEGs) (adjusted *p* < 0.05) were selected based on the absolute log_2_ fold change obtained from DESeq2. Gene expression values were scaled by row (Z-score), and genes were ordered according to log_2_ fold change. Gene set enrichment analysis (GSEA) was performed using the clusterProfiler R package (v4.18.4) [30] in a pre-ranked manner. Genes were ranked based on log_2_ fold change obtained from DESeq2. The Hallmark gene sets from the Molecular Signatures Database (MSigDB, v2026.1.Hs) were obtained using the msigdbr R package (v26.1.0), and gene sets with a false discovery rate (FDR) < 0.05 were considered significantly enriched. Generative artificial intelligence (AI)-assisted tools were used to support code drafting/debugging. All analyses were performed by the authors, and all AI-assisted outputs were independently reviewed, verified, and edited by the authors.

### 2.6. Ligand–Target Interaction Analysis

To examine the effect of tumor–stromal interaction within the bone TME on transcriptional changes in tumor cells, we performed ligand–target interaction analysis using the NicheNet R package (v2.2.0), a computational framework that predicts ligand–target interactions [23]. In a xenograft mouse model, human-derived genes originate from tumor cells, whereas mouse-derived genes originate from host stromal cells. To analyze signaling from host stromal cells to tumor cells, mouse-derived genes were regarded as candidate ligands (sender), and human-derived genes as candidate receptors and target genes (receiver). Mouse gene symbols were converted to their corresponding human orthologs using the homologene R package (v1.4.68.19.3.27). Target genes of interest were defined as genes significantly upregulated in human-derived tumor cells in the bone TME compared with the non-bone TME (adjusted *p* < 0.05 and log_2_ fold change > 0). All expressed genes in tumor cells were used as background genes. Candidate ligands were defined as genes that were significantly upregulated in mouse-derived stromal cells in the bone TME compared with the non-bone TME (adjusted *p* < 0.05 and log_2_ fold change > 0) and were present in the NicheNet ligand-receptor network. Ligand activity was inferred using the predict_ligand_activities function. Area under the precision-recall curve (AUPR) scores were used to visualize prioritized candidate ligands and evaluate the overall predictive performance of each ligand. For ligand–target visualization, top-ranked ligands based on Pearson correlation coefficients between predicted target genes and the observed gene set of interest were used to extract weighted ligand–target links from the NicheNet ligand–target matrix. Regulatory potential scores between candidate ligands and predefined target genes of interest were visualized as heatmaps. Ligand–target links were integrated with ligand-receptor interactions from the NicheNet prior network to construct ligand-receptor-target networks. Then, networks centered on a selected ligand and target genes were generated to illustrate key signaling relationships, and visualized using Cytoscape (v3.10.3).

### 2.7. Statistical Analysis

For comparisons between two groups in animal and IHC analyses, the Shapiro–Wilk test was used to evaluate whether the data were normally distributed, followed by either Student’s *t*-test or the Mann–Whitney *U* test. Tumor volume curves were analyzed using a linear mixed-effects model fitted with the lme4 (v1.1.37) and lmerTest (v3.2.1) R packages using restricted maximum likelihood estimation. Group, time, and their interaction were included as fixed effects, with mouse included as a random intercept. *p*-values were calculated using Satterthwaite’s approximation for degrees of freedom. Statistical significance was set at *p* < 0.05. All statistical analyses were performed using R for Windows version 4.5.0 (The R Foundation for Statistical Computing, Vienna, Austria) as implemented in RStudio 2026.01.1 (Posit, Boston, MA, USA).

## 3. Results

### 3.1. Bone Tumor Microenvironment Is Associated with Tumor Enlargement Despite Reduced Tumor Cell Proliferation in Meningioma Xenograft Models

**Figure 1:**
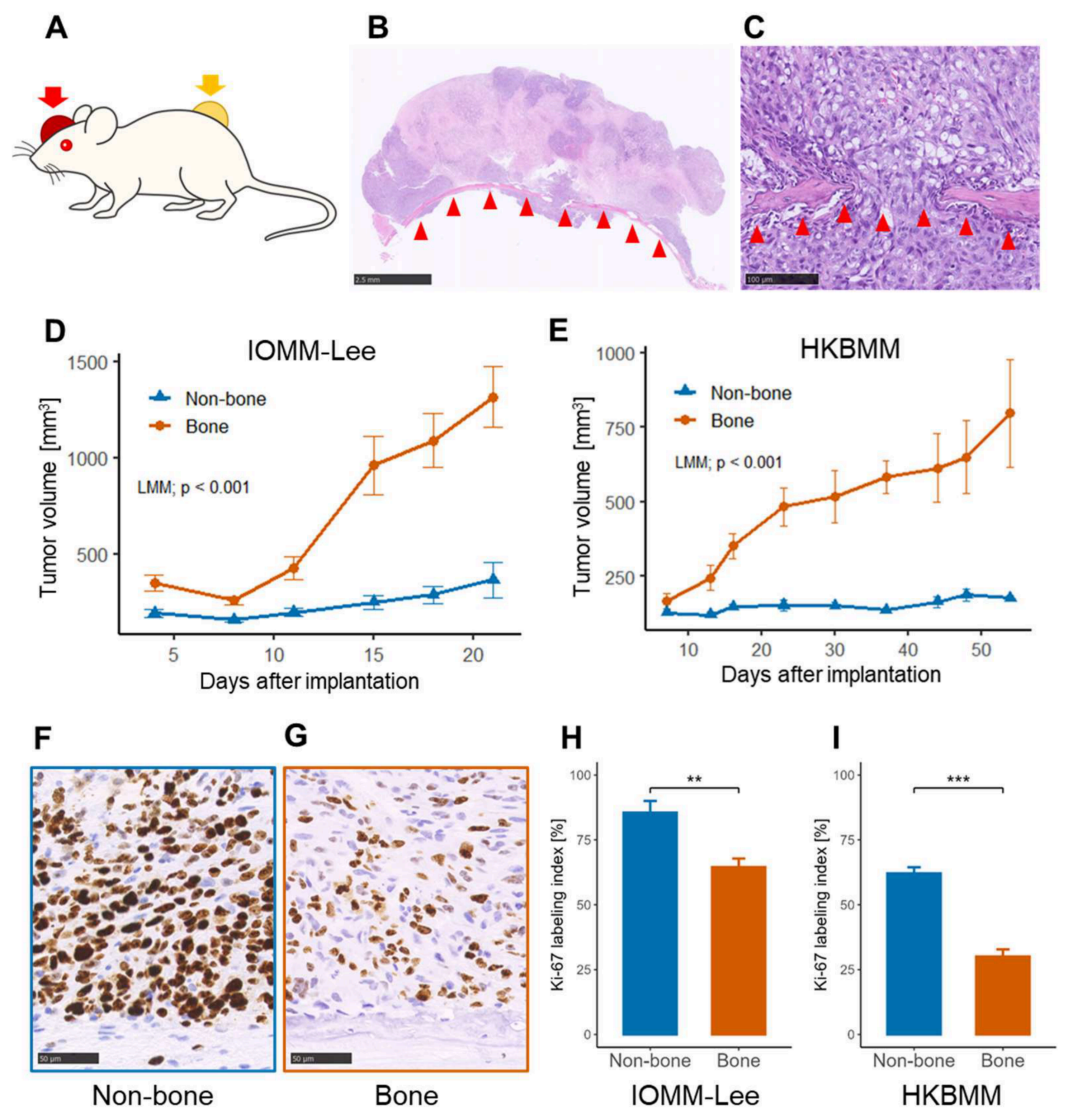
Analysis of tumor volume and tumor cell proliferation in the bone tumor microenvironment using meningioma xenograft models. (**A**) Schematic illustration of the xenograft model. Cell lines were subcutaneously implanted over the calvarial bone (bone tumor microenvironment (TME); red arrow) and into the dorsal skin (non-bone TME; yellow arrow) within the same mouse. (**B**) Representative low-magnification hematoxylin and eosin (H&E)-stained image of an IOMM-Lee tumor over the calvarial bone. Arrowheads indicate the tumor-bone interface. Scale bar, 2.5 mm. (**C**) Representative high-magnification H&E-stained image of an IOMM-Lee tumor over the calvarial bone. Tumor invasion was accompanied by bone destruction (arrowheads). Scale bar, 100 μm. (**D**,**E**) Growth curves of IOMM-Lee (**D**) and HKBMM (**E**) tumors in the non-bone and bone TMEs. Data are presented as mean ± SEM. Linear mixed-effects model (LMM) was used for analysis (IOMM-Lee: *p* < 0.001, HKBMM: *p* < 0.001). (**F**,**G**) Representative images of immunohistochemical (IHC) staining for Ki-67 in IOMM-Lee tumors from non-bone (**F**) and bone (**G**) TMEs. Scale bars, 50 μm. (**H**,**I**) Quantification of Ki-67 labeling index in IOMM-Lee (**H**) and HKBMM (**I**) tumors from non-bone and bone TMEs. Data are presented as mean ± SEM. ** *p* < 0.01, *** *p* < 0.001.

To investigate the impact of the bone TME on meningioma growth, we established a mouse xenograft model in which human malignant meningioma cell lines (IOMM-Lee and HKBMM) were implanted. Each cell line was subcutaneously implanted over the calvarial bone (bone TME) and into the dorsal skin (non-bone TME) within the same mouse (*n* = 5 per cell line) (Figure 1A). In the bone TME, tumor invasion into the calvarial bone accompanied by bone destruction was observed (Figure 1B,C). Tumor volume was significantly greater in the bone TME than in the non-bone TME in both the IOMM-Lee (Figure 1D; *p* < 0.001) and HKBMM (Figure 1E; *p* < 0.001) models. To assess tumor cell proliferation, we performed IHC staining for Ki-67 and quantified the labeling index (Figure 1F,G). Despite increased tumor volume, the Ki-67 labeling index was significantly lower in the bone TME than in the non-bone TME in both cell lines (Figure 1H,I).

### 3.2. Bone TME Is Associated with Distinct Transcriptional States in Meningioma Cells

**Figure 2:**
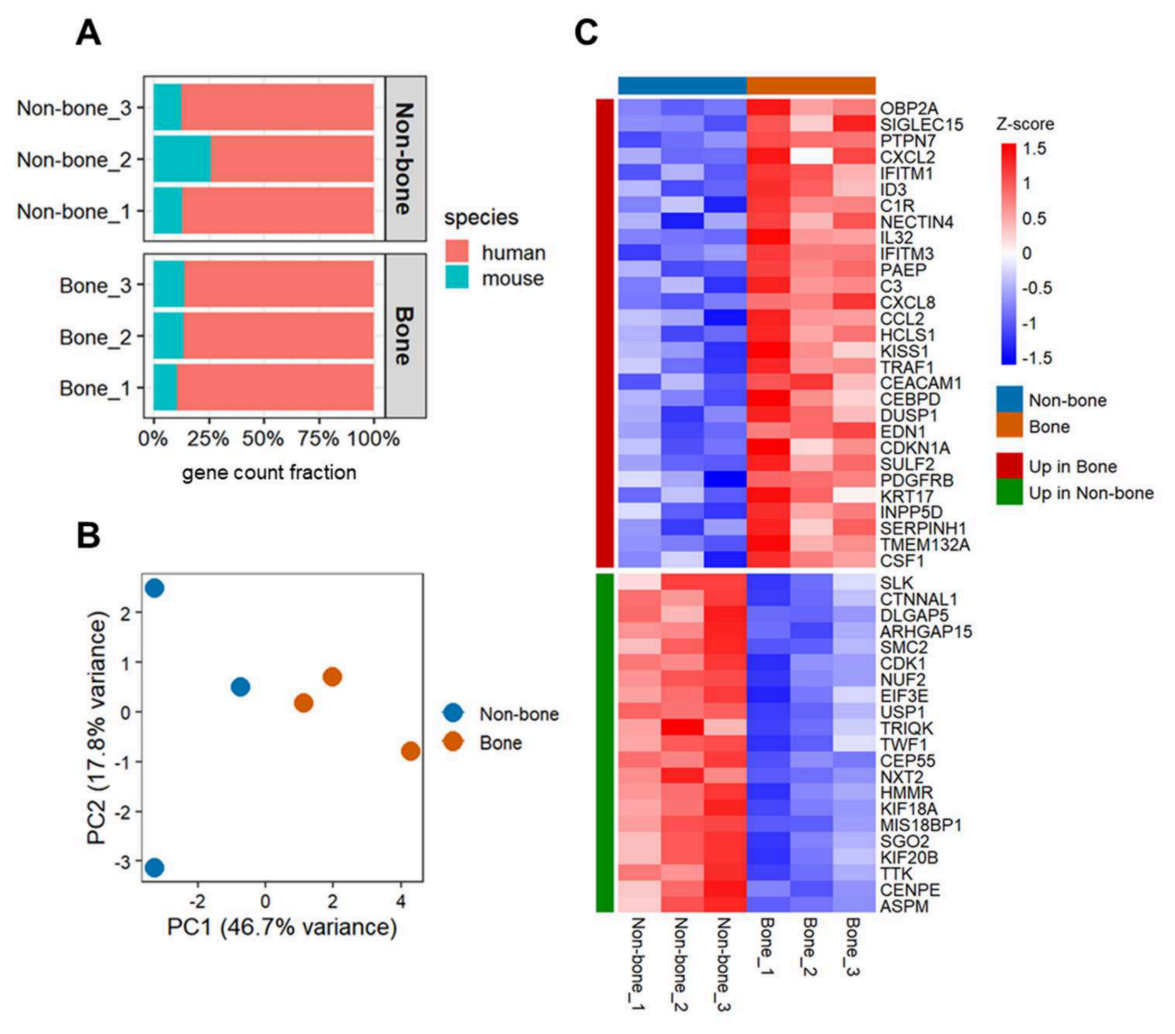
Transcriptomic differences between tumors derived from the bone and non-bone tumor microenvironments. (**A**) Proportion of sequencing reads assigned to human (tumor cell-derived) and mouse (stromal cell-derived) genomes in each sample based on raw counts quantified by HTSeq. (**B**) Principal component analysis (PCA) of human-derived transcripts. PC1, the first principal component; PC2, the second principal component. (**C**) Heatmap of the top 50 differentially expressed genes (DEGs) in human-derived transcripts between the bone and non-bone TMEs (DEGs: adjusted *p* < 0.05 and |log_2_ fold change| > 1). Expression values are shown as Z-scores.

To explore the molecular changes associated with the differences in these phenotypes between tumors derived from the bone and non-bone TMEs, we performed RNA-seq using the IOMM-Lee mouse model (*n* = 3). To distinguish tumor- and host-derived reads, sequencing reads were separated into human and mouse components. Most reads were assigned to the human genome, with a smaller proportion assigned to the mouse genome, consistent with effective separation of tumor and stromal compartments (Figure 2A). Focusing on human-derived transcripts, principal component analysis (PCA) showed clear separation between tumors from the bone and non-bone TMEs along the first principal component (Figure 2B). Heatmap visualization of the top 50 differentially expressed genes (DEGs) revealed distinct transcriptional differences between tumors from the bone and non-bone TMEs (Figure 2C). Genes upregulated in the bone TME predominantly included inflammation-related genes, such as chemokines and interferon-responsive genes. In contrast, genes upregulated in the non-bone TME predominantly included cell proliferation-related genes, such as cell cycle and cell division genes.

### 3.3. Bone TME Is Associated with Inflammatory Transcriptional Programs in Meningioma Cells

**Figure 3:**
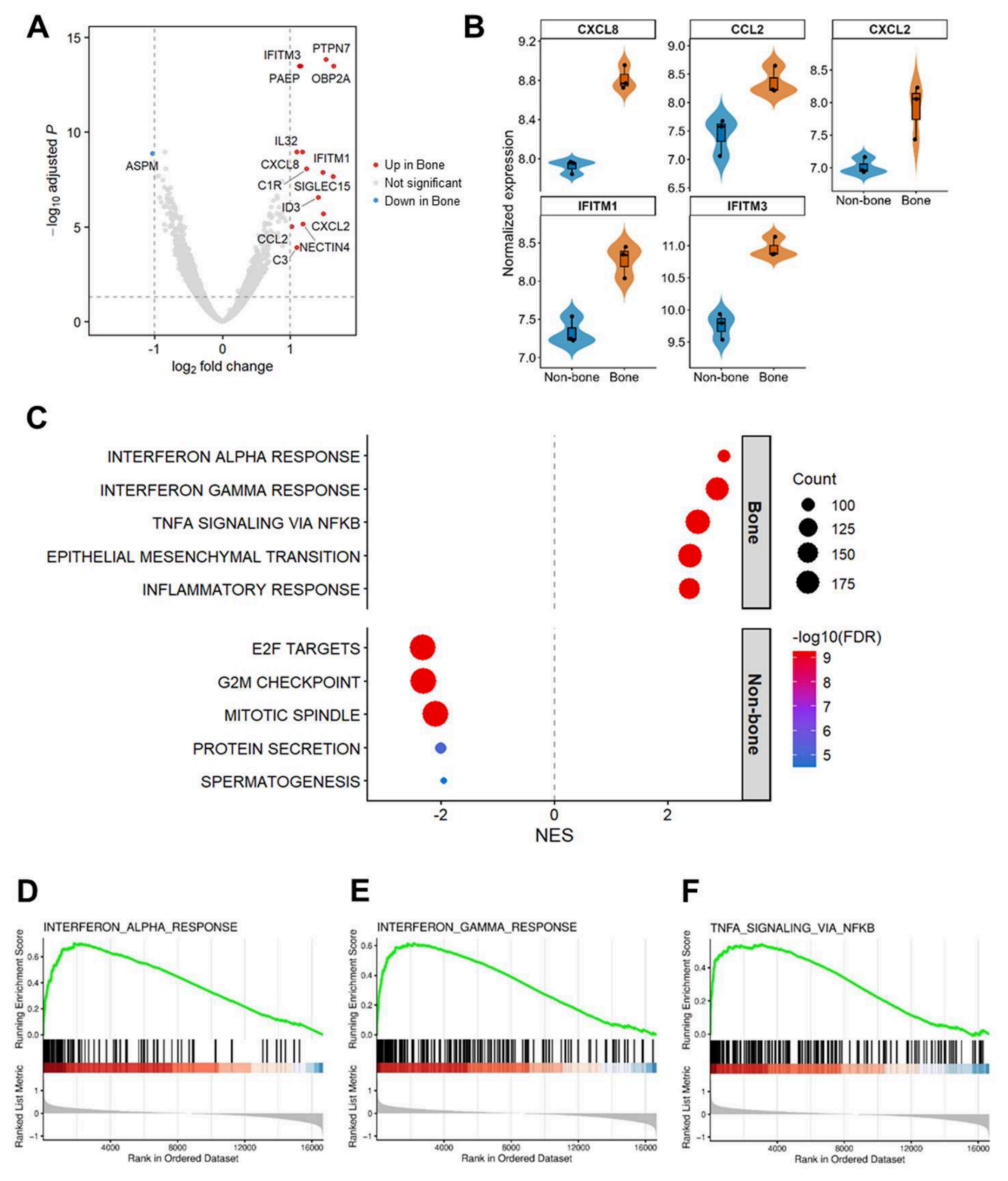
Enrichment of inflammation-related signaling in tumor cells from the bone tumor microenvironment. (**A**) Volcano plot highlighting differentially expressed genes (DEGs) in human-derived transcripts between the non-bone and bone tumor microenvironments (TMEs) (DEGs: adjusted *p* < 0.05 and |log_2_ fold change| > 1). Red, blue, and gray dots indicate genes upregulated in the bone TME, genes downregulated in the bone TME, and non-significant genes, respectively. (**B**) Violin plots showing normalized expression levels of representative inflammation-related genes (*CXCL8*, *CCL2*, *CXCL2*, *IFITM1*, and *IFITM3*) in tumor cells from the non-bone and bone TMEs. (**C**) Gene set enrichment analysis (GSEA) of Hallmark gene sets based on genes ranked by log_2_ fold change. The top five positively and negatively enriched gene sets ranked by normalized enrichment score (NES) are shown. Dot size indicates the number of contributing genes, and color indicates -log_10_(FDR), showing statistical significance. Positive NES corresponds to enrichment in the bone TME, whereas negative NES corresponds to enrichment in the non-bone TME. (**D**–**F**) Representative GSEA enrichment plots for interferon (IFN)-α response (**D**), IFN-γ response (**E**), and tumor necrosis factor (TNF)-α signaling via nuclear factor-kappa B (NF-κB) (**F**) pathways.

A volcano plot of DEGs revealed that multiple inflammation-related genes, including *CXCL8*, *CCL2*, and *CXCL2* (chemokines), as well as *IFITM1* and *IFITM3* (interferon-related genes), were significantly upregulated in tumor cells from the bone TME (Figure 3A). Violin plots showed that the normalized expression levels of these representative genes were higher in tumor cells from the bone TME than in those from the non-bone TME (Figure 3B). To identify the biological pathways associated with these transcriptional changes, we performed gene set enrichment analysis (GSEA). GSEA revealed enrichment of inflammatory signaling pathways in tumor cells from the bone TME, including interferon (IFN)-α response, IFN-γ response, and tumor necrosis factor (TNF)-α signaling via nuclear factor-kappa B (NF-κB) (Figure 3C–F). In contrast, cell cycle-related pathways, including E2F targets and G2M checkpoint, were enriched in tumor cells from the non-bone TME (Figure 3C).

### 3.4. Stromal IL1B Is a Candidate Regulator of Inflammatory Transcriptional Programs in Meningioma Cells Within the Bone TME

**Figure 4:**
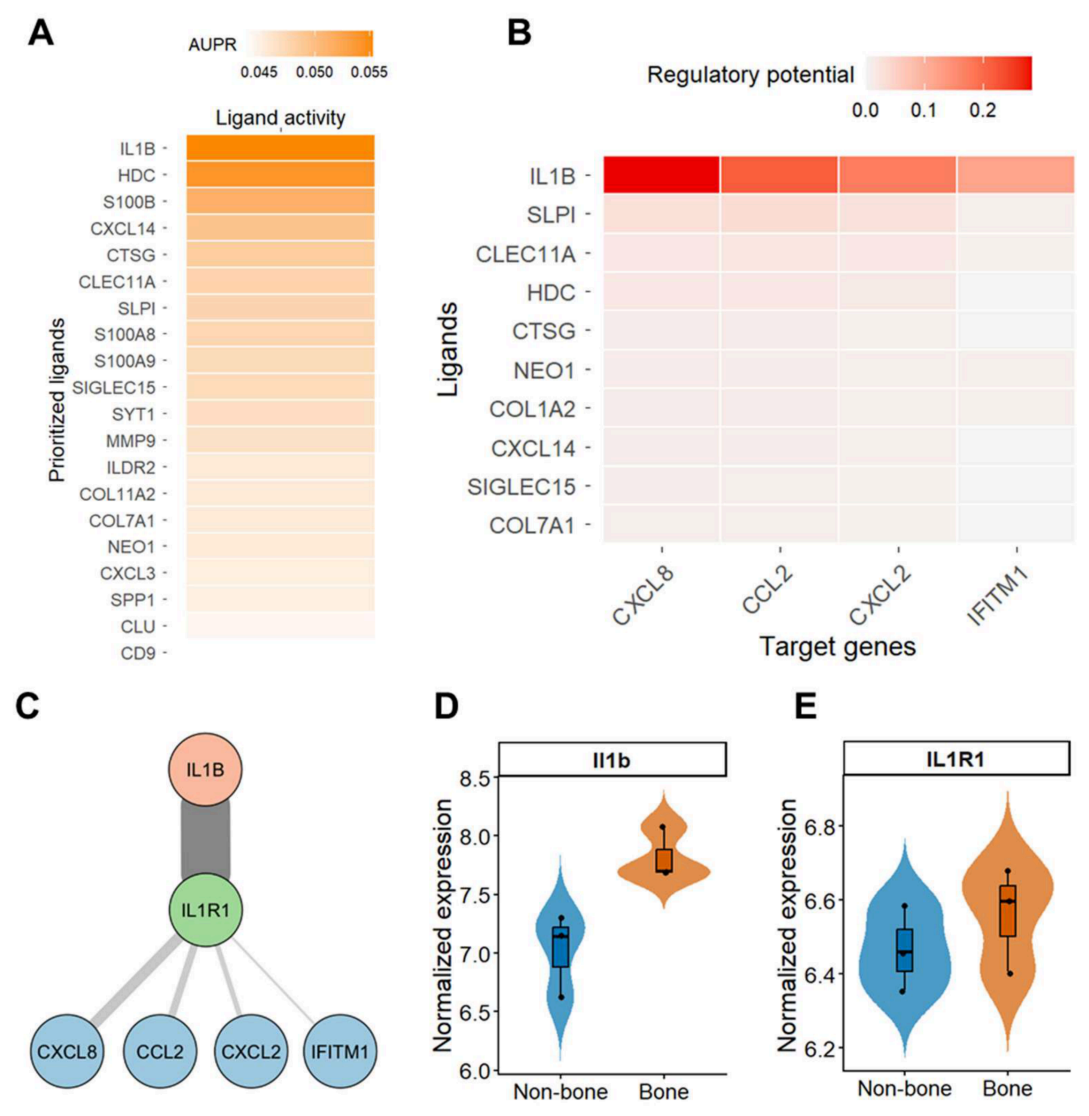
Identification of stromal IL1B as a candidate regulator of inflammation-related gene expression in the bone tumor microenvironment. (**A**–**C**) Ligand–target interaction analysis using NicheNet. (**A**) Ligand activity of prioritized stromal cell-derived ligands ranked based on area under the precision-recall curve (AUPR) scores. (**B**) Predicted regulatory potential of candidate ligands on selected target genes in tumor cells. (**C**) Schematic representation of the ligand-receptor-target network centered on *IL1B*, including *IL1R1* and selected target genes. Edge thickness indicates the predicted regulatory potential. (**D**) Violin plot showing normalized expression levels of *Il1b* in stromal cells from the non-bone and bone tumor microenvironments (TMEs). (**E**) Violin plot showing normalized expression levels of *IL1R1* in tumor cells from the non-bone and bone TMEs.

To further investigate bone TME-derived signaling associated with the upregulation of key genes in tumor cells, we performed ligand–target interaction analysis using NicheNet. The analysis modeled interactions between mouse stromal cell-derived ligands and human tumor cell-derived receptors. Differential ligand activity analysis identified candidate ligands derived from stromal cells that may regulate gene expression in tumor cells under the bone TME. Among these, *IL1B* was ranked as one of the top ligands based on AUPR scores, along with other candidates such as *HDC*, *S100B*, and *CXCL14* (Figure 4A). Furthermore, we assessed the potential regulatory effects of these ligands on key upregulated genes, including *CXCL8*, *CCL2*, *CXCL2*, *IFITM1*, and *IFITM3*. *IL1B* showed strong predicted regulatory potential on *CXCL8*, *CCL2*, *CXCL2*, and *IFITM1*, whereas other candidate ligands exhibited weaker regulatory effects (Figure 4B). Although *IFITM3* was included among the predefined target genes, regulatory effects on *IFITM3* were limited across these candidate ligands. We also visualized the ligand-receptor-target network centered on *IL1B*. A simplified network model illustrated that *IL1B* was predicted to interact with its receptor *IL1R1*, and to regulate multiple downstream target genes, including *CXCL8*, *CCL2*, *CXCL2*, and *IFITM1* (Figure 4C). *IL1B* (*Il1b* in the mouse ortholog) and *IL1R1* encode the interleukin-1 beta (IL-1β) and interleukin-1 receptor type 1 (IL1R1) protein, respectively. To examine the potential involvement of IL-1β/IL1R1 signaling in the bone TME, we assessed the expression of *Il1b* in mouse stromal cells and its receptor *IL1R1* in human tumor cells. The normalized expression level of *Il1b* was significantly higher in stromal cells from the bone TME than in those from the non-bone TME (Figure 4D). *IL1R1* was consistently expressed in tumor cells from both the bone and non-bone TMEs (Figure 4E).

### 3.5. Macrophages Express IL-1β and Meningioma Cells Show NF-κB-Related Phosphorylation Within the Bone TME

**Figure 5:**
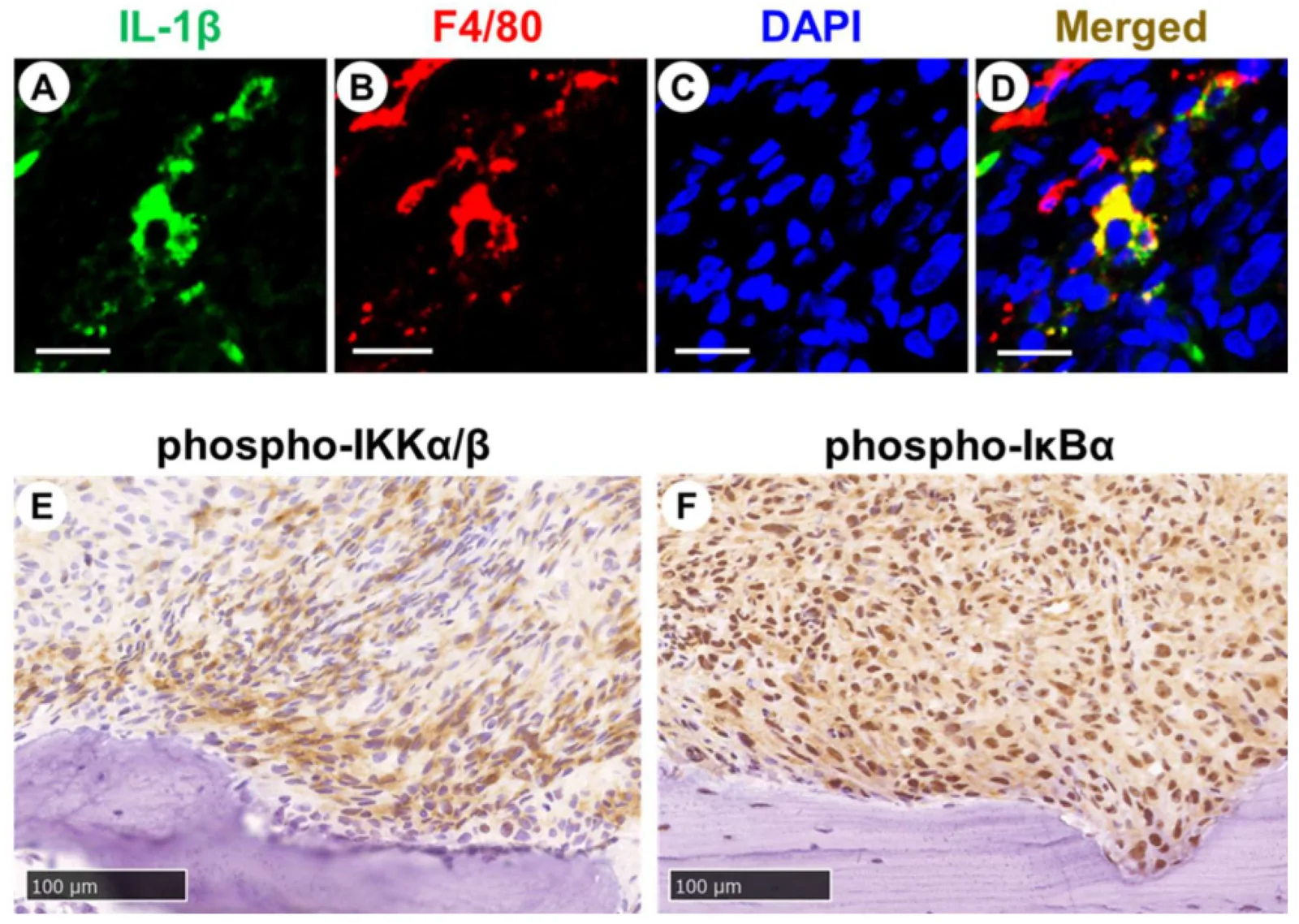
Multiplex immunofluorescence (IF) staining for IL-1β and F4/80, and immunohistochemical (IHC) staining for phospho-IKKα/β and phospho-IκBα. (**A**–**D**) Representative images of multiplex IF staining. (**A**) IL-1β-positive cells were visualized with FITC (green). (**B**) F4/80-positive cells were visualized with TRITC (red). (**C**) Nuclei were counterstained with DAPI (blue). (**D**) The merged image of (**A**–**C**). Scale bars, 20 μm. (**E**,**F**) Representative images of IHC staining for phospho-IKKα/β (**E**) and phospho-IκBα (**F**). Scale bar, 100 μm.

IL-1β is predominantly produced by myeloid-lineage cells, including macrophages [31,32]. To further examine its cellular source, we performed multiplex IF staining for IL-1β and F4/80, a murine pan-macrophage marker. IL-1β-positive cells and F4/80-positive cells were visualized with FITC (Figure 5A) and TRITC (Figure 5B), respectively. Nuclei were counterstained with DAPI (Figure 5C). The merged image showed co-localization of IL-1β and F4/80 positivity (Figure 5D). IL-1β has been shown to activate NF-κB signaling through IL1R1, thereby inducing multiple chemokines [33,34,35]. To examine whether the transcriptomic and ligand–target predictions were histologically concordant with NF-κB-related signaling within the bone TME, we also performed IHC staining for phosphorylated inhibitor of nuclear factor kappa-B kinase subunit alpha/beta (phospho-IKKα/β) and phosphorylated inhibitor of nuclear factor kappa-B alpha (phospho-IκBα). In the bone TME, tumor cells adjacent to calvarial bone were positive for both phospho-IKKα/β (Figure 5E) and phospho-IκBα (Figure 5F).

## 4. Discussion

In the context of bone invasion and bone metastasis, the bone TME has been recognized as a favorable “soil” that supports tumor progression through dynamic tumor–stromal interactions [15,16,36]. The bone TME has been shown to promote tumor growth accompanied by increased tumor cell proliferation in certain types of cancer, such as breast and prostate cancer [15,16]. In this study, we demonstrated that the bone TME was associated with tumor enlargement in a malignant meningioma xenograft model. However, tumor cell proliferation assessed by Ki-67 IHC was significantly reduced in the bone TME, in contrast to observations in other cancer types [15,16]. The phenotypes of increased tumor volume and reduced Ki-67 labeling were reproduced in two independently established meningioma cell lines, suggesting that they were not specific to a single cell line within the present experimental model. This discrepancy suggests that tumor enlargement in the bone TME may not be simply explained by enhanced tumor cell proliferation. In general, tumor enlargement in vivo can reflect not only proliferative activity but also additional biological processes, including local invasion, tissue remodeling, and other microenvironmental support [37,38]. Ki-67 labeling reflects the fraction of proliferating tumor cells at the time of tissue sampling, whereas tumor volume represents the cumulative result of multiple processes, including tumor cell proliferation and survival as well as contributions from stromal and extracellular components. From a tumor cell-intrinsic perspective, attenuation of apoptosis-related signaling or enhancement of survival signaling could potentially contribute to tumor enlargement independently of proliferative activity. From a stromal perspective, microenvironment-derived soluble factors or proteases could modulate death pathways in tumor cells. For example, matrix metalloproteinase 7-mediated cleavage of Fas ligand has been reported to modify Fas-mediated apoptosis and promote tumor cell survival [39]. Therefore, stromal cell-derived proteolytic activity could potentially influence tumor cell survival. Because the present study did not quantitatively assess the relative tumor cell and stromal fractions or evaluate apoptosis-related proteins, protease activity, or cell-death signaling, the mechanisms underlying the apparent discrepancy between increased tumor volume and reduced Ki-67 labeling remain to be determined. Based on these findings, we next investigated bone TME-specific signaling associated with tumor enlargement in this context.

Previous studies have demonstrated that the bone TME can substantially change tumor cell transcriptional states in bone metastases across multiple cancer types, such as renal cell carcinoma [17] and breast carcinoma [40], as well as in bone-invasive pituitary neuroendocrine tumors [41]. In this study, we showed that the bone TME was associated with distinct transcriptional changes in tumor cells, using a meningioma xenograft mouse model with species-specific transcriptomic analysis. We further showed that inflammation-related genes were predominantly upregulated in the bone TME, whereas cell proliferation-related genes were predominantly upregulated in the non-bone TME. These findings are consistent with our observation that tumor cell proliferation assessed by Ki-67 IHC was reduced in the bone TME. In conventional bulk RNA-seq of tumor tissues, transcriptomic signals from tumor and stromal cells are mixed, making it difficult to assign signaling changes to specific cellular compartments. In the present xenograft framework, human-derived reads were used to define tumor cell transcriptional states, whereas mouse-derived reads were used to identify stromal cell-derived candidate ligands. This design allowed us to model stromal cell-to-tumor cell signaling, rather than simply identifying pathways enriched in bulk tumor tissue.

We also showed that inflammation-related genes, including *CXCL8*, *CCL2*, *CXCL2*, *IFITM1*, and *IFITM3*, were significantly upregulated in tumor cells from the bone TME. GSEA revealed enrichment of inflammation-related pathways, including IFN-α, IFN-γ, and TNF-α/NF-κB signaling pathways, whereas cell cycle-related pathways, including E2F targets and G2M checkpoint, were negatively enriched in the bone TME. The concomitant enrichment of inflammatory pathways and negative enrichment of cell cycle-related pathways may reflect a shift in tumor cell state from a highly proliferative phenotype toward an inflammation-associated adaptive phenotype in the bone TME. Within the TME, IFN-α, IFN-γ, and TNF-α/NF-κB signaling pathways can facilitate tumor progression through tumor cell adaptation, inflammatory remodeling, and therapy resistance [33,42,43,44]. The expression of *CXCL8*, *CCL2*, and *CXCL2* is known to be induced by TNF-α/NF-κB signaling [33], whereas *IFITM1* and *IFITM3* are representative interferon-stimulated genes whose expression is known to be induced by IFN-α and IFN-γ signaling [45]. C-X-C motif chemokine ligand 8 (CXCL8), C-C motif chemokine ligand 2 (CCL2), and CXCL2 are chemokines involved in the recruitment of neutrophils and monocytes [46]. These chemokines have also been shown to promote tumor progression and to be associated with poor prognosis in several cancers [33]. Interferon-induced transmembrane protein 1 (IFITM1) and IFITM3 are molecules involved in the immune response to viral infection [45]. These molecules have also been shown to be highly expressed in various cancers, such as breast cancer, gastric cancer, and glioblastoma, and to be associated with tumor cell invasion, metastasis, and therapy resistance [47,48,49,50]. In the present model, the coexistence of tumor enlargement and reduced tumor cell proliferation suggests that bone TME-associated tumor enlargement is not simply proliferation-dependent, but may involve additional microenvironmental processes. Our findings indicate that the bone TME was associated with inflammatory transcriptional programs in tumor cells, concurrent with tumor enlargement despite reduced tumor cell proliferation. We next hypothesized that this inflammatory transcriptional state associated with tumor enlargement may be involved in stromal cell-to-tumor cell signaling within the bone TME.

In the present ligand–target analysis, *IL1B* showed the strongest ligand activity among stromal cell-derived candidate ligands and high predicted regulatory potential for key inflammation-related genes, including *CXCL8*, *CCL2*, *CXCL2*, and *IFITM1*. In contrast, other candidate ligands showed relatively limited regulatory effects. These findings support stromal *IL1B* as a prominent candidate regulator of the inflammatory transcriptional program in tumor cells within the bone TME. Because the predicted regulatory effect of *IL1B* on *IFITM3* was limited compared with other key target genes, additional signaling pathways beyond IL-1β/IL1R1 signaling may contribute to the induction of *IFITM3*. IL-1β is a pro-inflammatory cytokine that primarily signals through IL1R1 and activates downstream pathways including NF-κB signaling, thereby inducing multiple chemokines [33,34,35]. Based on our findings, IL-1β/IL1R1 signaling may contribute to the upregulation of *CXCL8*, *CCL2*, and *CXCL2* through the activation of NF-κB signaling in meningioma cells within the bone TME. NF-κB signaling has also been shown to cooperate with interferon pathways and contribute to the induction of interferon-related gene expression [34,51]. Although IFITM family genes are primarily induced by interferon signaling [45], IL-1β/IL1R1 signaling may indirectly contribute to their upregulation through NF-κB signaling and potential crosstalk with interferon pathways in the bone TME. We further demonstrated that *Il1b* was significantly upregulated in stromal cells within the bone TME, while *IL1R1* was consistently expressed in tumor cells regardless of the microenvironment. This expression pattern suggests that meningioma cells retain the capacity to respond to IL-1β, and that increased ligand availability in the bone TME may enhance downstream signaling. IL-1β has also been shown to be involved in osteoclast activation and bone resorption [52,53]. Increased IL-1β expression in stromal cells within the bone TME could therefore also be relevant to osteoclast-mediated bone destruction. This possibility is consistent with our histological observation of tumor invasion accompanied by bone destruction, although osteoclast activity was not directly examined in the present study.

Macrophages are known to be major producers of IL-1β [31,32]. By multiplex IF staining, we found that F4/80-positive macrophages expressed IL-1β in the bone TME of meningioma. This finding suggests that macrophages are one of the cellular sources of IL-1β in this context. In addition, we found that meningioma cells were positive for both phospho-IKKα/β and phospho-IκBα in the bone TME by IHC staining. Phosphorylation of these canonical NF-κB regulators is consistent with pathway activation, suggesting involvement of NF-κB signaling in meningioma cells within the bone TME. Taken together, these findings support the possibility that macrophage-derived IL-1β is involved in NF-κB-related inflammatory signaling and associated transcriptional programs in meningioma cells within the bone TME.

This study has some limitations. Tumor volume was estimated from external measurements, and tumor cell density and stromal or extracellular components were not separately quantified. Furthermore, apoptosis-related proteins, protease activity, and cell-death signaling were not evaluated. Therefore, the relative contributions of tumor cell-intrinsic mechanisms and stromal components to the increased tumor volume despite reduced Ki-67 labeling cannot be determined. Although tumor invasion into the calvarial bone accompanied by bone destruction was observed in the present model, the possibility that cell injection-related microinjury contributed to bone destruction cannot be entirely excluded. In the present heterotopic model, the tumors over the calvarial bone developed in the supraosseal compartment. Therefore, unlike an orthotopic intracranial meningioma model, this model could not reproduce the entire native intracranial microenvironment, including the dura mater, arachnoid, cerebrospinal fluid, and brain parenchyma [24]. Our model is supported by the concordance among species-specific transcriptomic findings, computational ligand–target predictions, and the histological localization of the relevant cellular and signaling components. However, each line of evidence remains associative. NicheNet analysis predicts potential ligand–target relationships but does not establish direct signaling; the colocalization of IL-1β and F4/80 identifies macrophages as a possible cellular source of IL-1β but does not demonstrate its functional activity; and immunoreactivity for phospho-IKKα/β and phospho-IκBα is consistent with NF-κB-related signaling but does not establish that it is dependent on IL-1β. Furthermore, no interventional experiments were performed to determine whether IL-1β/IL1R1 signaling is necessary for the observed transcriptional changes or tumor enlargement. In addition, although tumor enlargement and reduced tumor cell proliferation were observed in two meningioma cell lines, the transcriptomic and ligand–target analyses were performed using the IOMM-Lee model. Therefore, the inflammatory transcriptional states and candidate stromal IL-1β-related signaling identified in this study cannot currently be generalized to HKBMM or other meningioma models. Although multiplex IF staining for IL-1β and F4/80 and IHC staining for phospho-IKKα/β and phospho-IκBα were performed to examine whether transcriptomic and ligand–target predictions were histologically concordant with NF-κB-related signaling within the bone TME, comparative multiplex IF and IHC analyses between the bone and non-bone TMEs were not performed. The mechanisms linking the inflammatory transcriptional states to tumor enlargement, as well as the functional role of macrophage-derived IL-1β in stromal cell-to-tumor cell signaling, warrant further investigation.

## 5. Conclusions

Our findings indicate that the bone TME is associated with tumor enlargement despite reduced tumor cell proliferation and with inflammatory transcriptional programs in meningioma cells. Tumor–stromal interactions potentially involving macrophage-derived IL-1β may be associated with NF-κB-related inflammatory transcriptional changes and with tumor enlargement in the bone TME. These interactions may warrant further investigation as potential therapeutic targets for meningioma with bone involvement.

## Data Availability

All RNA-seq data generated in this study have been deposited in the NCBI Gene Expression Omnibus under accession number GSE336430. The codes for RNA-seq data processing and downstream analyses are available from the corresponding author upon request.
